# Differential molecular mechanisms of substrate recognition by selenium methyltransferases, INMT and TPMT, in selenium detoxification and excretion

**DOI:** 10.1016/j.jbc.2023.105599

**Published:** 2023-12-28

**Authors:** Yasunori Fukumoto, Rin Kyono, Yuka Shibukawa, Yu-ki Tanaka, Noriyuki Suzuki, Yasumitsu Ogra

**Affiliations:** Graduate School of Pharmaceutical Sciences, Chiba University, Chiba, Japan

**Keywords:** selenium, enzyme mechanism, enzyme catalysis, metabolism, molecular dynamics, trimethylselenonium ion, indolethylamine *N*-methyltransferase, thiopurine *S*-methyltransferase, inductively coupled plasma mass spectrometry, fragment molecular orbital method

## Abstract

It is known that the recommended dietary allowance of selenium (Se) is dangerously close to its tolerable upper intake level. Se is detoxified and excreted in urine as trimethylselenonium ion (TMSe) when the amount ingested exceeds the nutritional level. Recently, we demonstrated that the production of TMSe requires two methyltransferases: thiopurine *S*-methyltransferase (TPMT) and indolethylamine *N*-methyltransferase (INMT). In this study, we investigated the substrate recognition mechanisms of INMT and TPMT in the *Se*-methylation reaction. Examination of the *Se*-methyltransferase activities of two paralogs of INMT, namely, nicotinamide *N*-methyltransferase and phenylethanolamine *N*-methyltransferase, revealed that only INMT exhibited *Se*-methyltransferase activity. Consistently, molecular dynamics simulations demonstrated that dimethylselenide was preferentially associated with the active center of INMT. Using the fragment molecular orbital method, we identified hydrophobic residues involved in the binding of dimethylselenide to the active center of INMT. The INMT-L164R mutation resulted in a deficiency in *Se*- and *N*-methyltransferase activities. Similarly, TPMT-R152, which occupies the same position as INMT-L164, played a crucial role in the *Se*-methyltransferase activity of TPMT. Our findings suggest that TPMT recognizes negatively charged substrates, whereas INMT recognizes electrically neutral substrates in the hydrophobic active center embedded within the protein. These observations explain the sequential requirement of the two methyltransferases in producing TMSe.

Selenium (Se) is an essential trace element in animals and belongs to the chalcogen group that also includes sulfur and tellurium. Se ingested at the nutritional level in a regular diet is metabolized and excreted as selenosugars in urine (1, 2, 3). However, Se has a narrow range of nutritional levels because its recommended dietary allowance differs from its tolerable upper intake level by only one order of magnitude (4). When the amount of Se ingested exceeds the nutritional level, the excess Se undergoes detoxification through methylation reactions and is excreted in urine in the trimethylated form, trimethylselenonium ion (TMSe) (1, 5, 6). Recently, we discovered that the production of TMSe requires two methyltransferases: thiopurine *S*-methyltransferase (TPMT) and indolethylamine *N*-methyltransferase (INMT) (7). TPMT catalyzes the first methylation step to produce methaneselenol. The second methylation step requires either TPMT or INMT as both methyltransferases are capable of producing dimethylselenide (DMSe). Finally, INMT mediates the third methylation step to produce TMSe. These findings have prompted further inquiry into the underlying rationale for the sequential requirement of the two methyltransferases in this specific order.

The INMT protein, initially identified as a thioether methyltransferase, was purified from mouse lungs and found to exhibit methyltransferase activity towards DMSe, dimethylsulfide (DMS), dimethyltelluride, and other compounds (8). *INMT* was initially cloned as mouse *TEMT* (9) and later identified in humans and rabbits as a gene that encodes methyltransferases responsible for the *N*-methylation of tryptamine and other compounds (10, 11). The structure of INMT with *S*-adenosylhomocysteine, but not its substrate recognition mechanism, has been reported (https://www.rcsb.org/structure/2A14). INMT has two paralogs within the human genome: nicotinamide *N*-methyltransferase (NNMT) and phenylethanolamine *N*-methyltransferase (PNMT). Together, INMT, NNMT, and PNMT form a family of methyltransferases referred to as the INMT family, which is conserved among vertebrates (11). The physiological substrates of NNMT and PNMT are nicotinamide and noradrenaline, respectively, and their cocrystal structures have been reported (12, 13). On the other hand, the *Se*-methyltransferase activities of NNMT and PNMT have yet to be elucidated.

TPMT has long been recognized as an *S*-methyltransferase involved in the metabolism of 6-mercaptopurine (6-MP), a chemotherapy drug for acute lymphocytic leukemia and inflammatory bowel disease (14, 15). The crystal structure of TPMT has been reported, and an Arg residue in the active center is suggested to be involved in the methylation of 6-MP (16). However, the endogenous substrate of TPMT has remained unknown, and the physiological importance of TPMT has been deliberated in the context of 6-MP metabolism, considering its potential influence on chemotherapeutic efficacy and toxicity. Our work and those of others have demonstrated that human TPMT methylates Se to aid in detoxification and excretion (7, 17). In addition, it has been observed that bacterial TPMT methylates Se, thereby facilitating the detoxification of environmental Se found in soil and water (18, 19). These findings strongly suggest that Se, which functions as both an essential nutrient and an environmental pollutant, is the endogenous substrate of TPMT.

Se is a crucial substrate for INMT and TPMT; however, the substrate recognition mechanisms involved in the methylation of Se by these enzymes remain a puzzle. In this study, we aim to investigate the substrate recognition mechanisms underlying the *Se*-methylation reaction in order to elucidate the mechanistic details of the different substrate specificities of TPMT and INMT.

## Results

### INMT, but not NNMT or PNMT, methylates Se to produce TMSe

**Figure 1:**
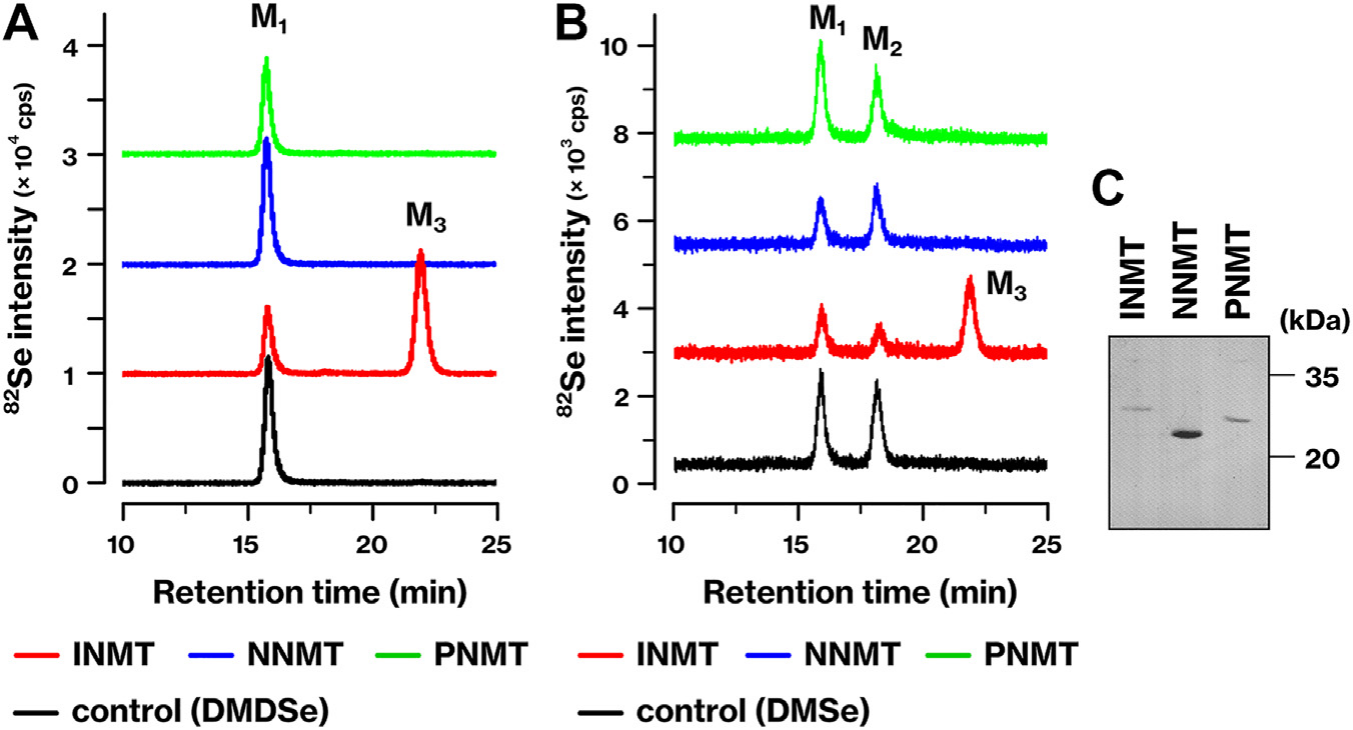
**Exclusive methylation of selenium (Se) by INMT producing trimethylselenonium ion (TMSe).***A* and *B*, recombinant proteins of human INMT, NNMT, and PNMT were tested for their ability to methylate the monomethylated (*A*) or dimethylated (*B*) form of Se. The reaction mixture included dimethyldiselenide (DMDSe) (*A*) or dimethylselenide (DMSe) (*B*), glutathione (GSH), and methyltransferases. The resulting reaction products were analyzed using LC‒ICP-MS. Control experiments were conducted in the absence of methyltransferases. Data represent the results of more than three independent experiments (*A* and *B*). Counts per second (cps) of ^82^Se are shown. M_1_ represents methaneseleninic acid (MSA), M_2_ represents dimethylselenoxide (DMSeO), and M_3_ represents TMSe. *C*, recombinant proteins of human INMT, NNMT, and PNMT were purified to near homogeneity and then separated using SDS-PAGE, transferred onto a PVDF membrane, and stained with Coomassie Brilliant Blue. INMT, indolethylamine *N*-methyltransferase; NNMT, nicotinamide *N*-methyltransferase; PNMT, phenylethanolamine *N*-methyltransferase; TMSe, trimethylselenonium ion.

The INMT family exhibits significant homology (Table 1). Human INMT shares 53% sequence identity and 67% similarity to NNMT while also displaying some degree of similarity to PNMT, albeit to a lesser extent. In contrast, INMT shows limited homology with TPMT. On the basis of these findings, we investigated the *Se*-methyltransferase activities of NNMT and PNMT. The *Se*-methylation reaction was analyzed using a hyphenated technique, high-performance liquid chromatography (HPLC) coupled with an inductively coupled plasma mass spectrometer (ICP-MS), referred to as LC‒ICP-MS, which enables the speciation of reaction products (1). In the elution profiles of the standard compounds (Fig. S1), the nonmethylated form of Se, selenite (M_0_), was eluted at 16.2 min (7). When selenite was mixed with glutathione (GSH), an additional peak appeared at 13.8 min, which may correspond to selenodiglutathione (GSSeSG). The mono-methylated form was detected along with the dimethylated form in the reaction mixture (Fig. 1*B*). In our previous study, we demonstrated that the dimethylated form, DMSe, was degraded by hydrogen peroxide treatment following the methylation reaction, leading to the production of the mono-methylated form (7).

**Table 1:** Paralogs of INMT in the human genome

Fukumoto, Kyono *et al.*, Table 1.

Indolethylamine *N*-methyltransferase (INMT) has two paralogs, nicotinamide *N*-methyltransferase (NNMT) and phenylethanolamine *N*-methyltransferase (PNMT), within the human genome. The table illustrates the levels of similarity and identity for human INMT, NNMT, PNMT, and thiopurine *S*-methyltransferase (TPMT) proteins. N.D., not determined.

We investigated the methyltransferase activities of NNMT and PNMT on Se. Human INMT, NNMT, and PNMT were expressed in bacteria and purified to nearly homogeneous levels (Fig. 1*C*). As previously reported (7), INMT methylated both the mono- and di-methylated forms of Se, leading to the formation of the trimethylated form, TMSe (Fig. 1, *A* and *B*). In contrast, neither NNMT nor PNMT exhibited methyltransferase activity towards the mono- or di-methylated form of Se, as evidenced by the similarity of the reaction products to those in the control reactions (Fig. 1, *A* and *B*). These findings indicate that although the methyltransferases in the INMT family share high identity and similarity, only INMT possesses the *Se*-methyltransferase activity.

### *In silico* analysis shows strong association of DMSe with INMT active center

**Figure 2:**
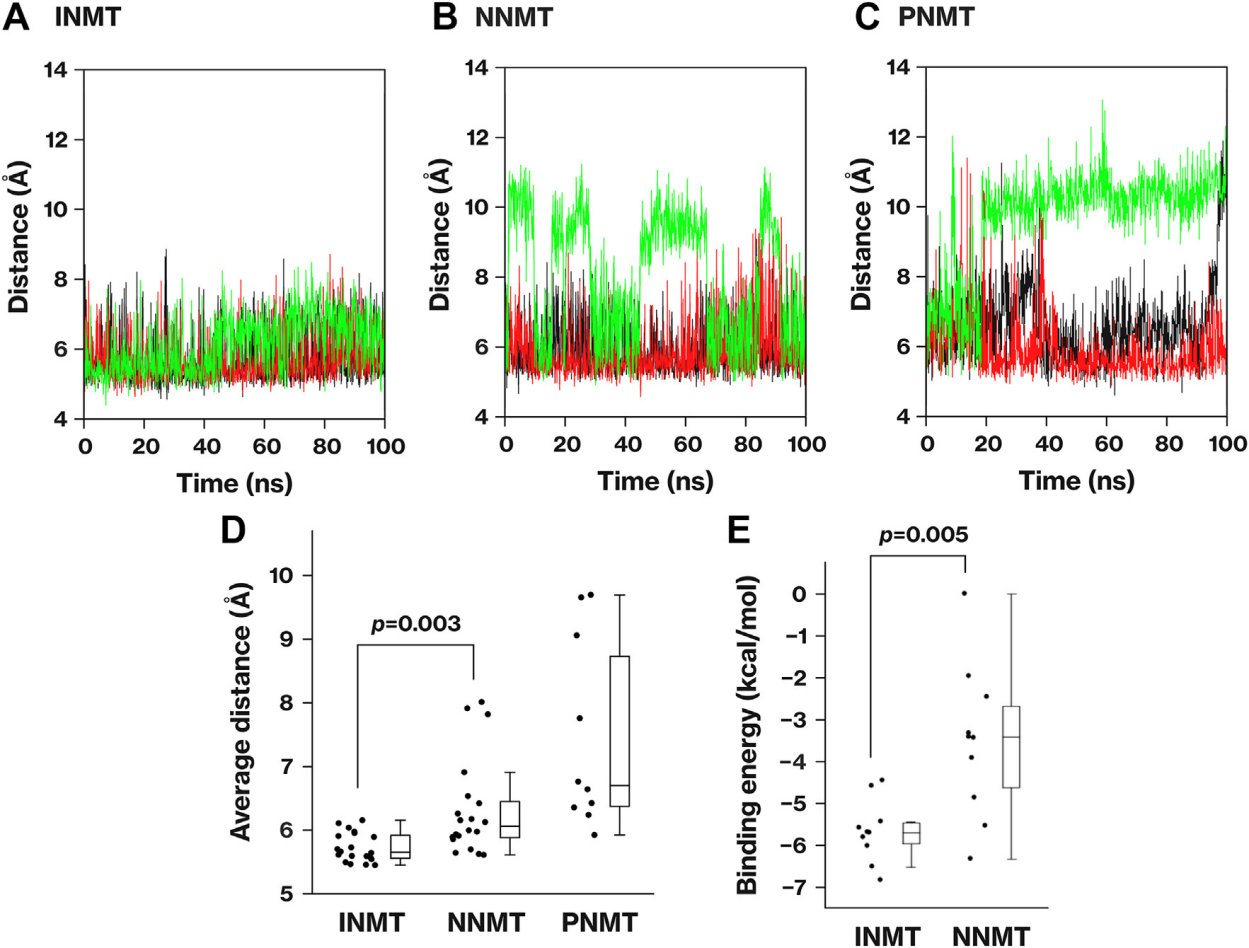
**Strong association of DMSe with the active center of the INMT protein *in silico*.***A*‒*C*, molecular dynamics (MD) simulations were performed to investigate the association of DMSe with the active centers of INMT (*A*), NNMT (*B*), and PNMT (*C*). DMSe was positioned within the active center of the respective methyltransferases, and the simulations were conducted using GROMACS. The distances between SAM and DMSe are displayed. Results of three independent simulations are presented, specifically from the first three simulations. *D*, the graph illustrates the average distance between SAM and DMSe. For INMT and NNMT, the simulation was repeated 20 times (*A* and *B*), whereas for PNMT, the simulation was repeated ten times. *E*, the binding energy (*ΔE*_*bind*_) between the methyltransferase/SAM dimer and DMSe was calculated using the FMO3-DFTB3/PCM<1> method. Ten trajectories from the MD simulations with small S-Se distances were selected from INMT and NNMT, and the corresponding averaged structures were generated. *p*-values were determined using Welch’s *t* test. DFTB, density functional tight binding; FMO, fragment molecular orbital; INMT, indolethylamine *N*-methyltransferase; NNMT, nicotinamide *N*-methyltransferase; PCM, polarizable continuum model; PNMT, phenylethanolamine *N*-methyltransferase; SAM, *S*-adenosylmethionine.

We conducted a molecular dynamics (MD) simulation to analyze the binding of Se compounds to the active centers of the INMT family. DMSe was selected as the compound for the MD simulation. In the methylation of the mono-methylated forms of Se, potential substrates may include various chemical forms such as methaneselenol and *Se*-methylglutathione selenopersulfide. However, in the methylation of the dimethylated form of Se, DMSe is the sole substrate. During the MD simulation, when DMSe was placed in the active center of INMT, the distance between *S*-adenosylmethionine (SAM) and DMSe was the shortest (Fig. 2, *A*‒*D*), indicating a strong association between INMT and DMSe. In NNMT, DMSe was also associated with the active center; however, the distance between SAM and DMSe was slightly but significantly longer than that in INMT. In PNMT, DMSe dissociated from the active center. An averaged structure was generated from the MD trajectory, and the binding energy of DMSe to the methyltransferases was estimated using the fragment molecular orbital (FMO) method. Binding energy calculations further supported the greater stability of the interaction between DMSe and INMT than that between DMSe and NNMT (Fig. 2*E*). These results demonstrate a correlation between the simulation results and the *in vitro* results.

### Association of DMSe with L164, Y204, and Y24 residues in INMT active center

**Figure 3:**
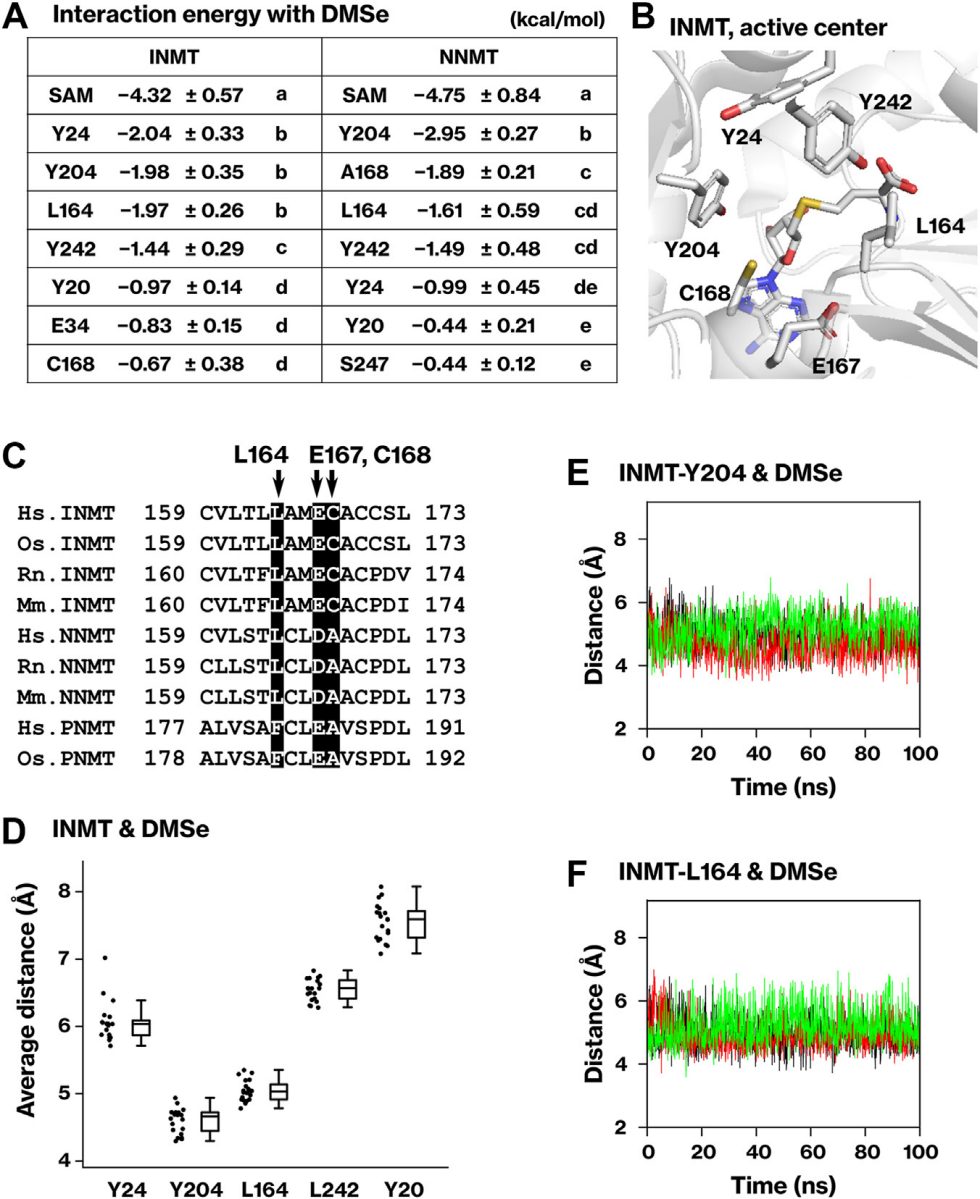
**Association of DMSe with L164, Y204, and Y24 residues in the active center of INMT.***A*, the interaction energy between DMSe and each residue of INMT or NNMT was calculated using the FMO2-DFTB3/PCM<1> method. The same averaged structures as in Figure 2*E* were utilized for the calculation. The table presents the results of ten averaged structures. Different letters indicate significant difference based on Tukey’s range test. *B*, a crystal structure of the INMT active site (PDB 2A14) is presented. *C*, multiple alignments of the INMT family were generated using Clustal Omega. Arrows indicate human INMT-L164, E167, and C168. The alignments of INMT-Y20, Y24, and Y242 are depicted in Fig. S2. Hs: human, Os: rabbit, Rn: rat, Mm: mouse. *D*, the average distance between DMSe and each residue of INMT was calculated from the 20 simulations in Figure 2. *E* and *F*, distances between DMSe and INMT-Y204 or L164 are displayed. The results of the first three simulations are shown. DFTB, density functional tight binding; FMO, fragment molecular orbital; INMT, indolethylamine *N*-methyltransferase; NNMT, nicotinamide *N*-methyltransferase; PCM, polarizable continuum model.

The mechanism by which DMSe associates with the active center of INMT was analyzed on the basis of the trajectory of the MD simulation. Pair interaction energy (PIE), also known as inter-fragment interaction energy, of the averaged structures was calculated using the FMO method. The PIEs represent the interaction between DMSe and each methyltransferase residue (20, 21). SAM played a significant role in the association of DMSe with the active center of both INMT and NNMT, exhibiting the highest binding energy (Fig. 3*A*). In the pair interaction energy decomposition analysis of the DMSe‒SAM interaction, the electrostatic interaction, electron dispersion, and solvation energies were determined to be −3.35 ± 0.50 kJ/mol, −1.39 ± 0.23 kJ/mol, and 0.45 ± 0.38 kJ/mol, respectively. One possible explanation is that the positive charge on the sulfur of SAM interacted with the dipole moment of DMSe. In the active center of INMT, hydrophobic residues Y24, Y204, L164, and Y242 interacted with DMSe (Fig. 3, *A* and *B*). Pair interaction energy decomposition analysis indicated that the majority of the interaction energy was due to electron dispersion (data not shown). These amino acid residues were conserved in NNMT but not in PNMT (Figs. 3*C* and S1), in agreement with the result of the MD simulation where the association of DMSe was least stable in PNMT (Fig. 2, *C* and *D*). In the MD simulation depicted in Figure 2, residues Y204 and L164 of INMT exhibited the shortest distance from DMSe and formed stable associations (Fig. 3, *D*‒F).

In the active center of NNMT, hydrophobic residues Y24, Y204, L164, and Y242 interacted with DMSe (Fig. 3*A*); however, the binding energy distribution varied. Among the amino acid residues listed in Figure 3*A*, only the pair of INMT-C168 and NNMT-A168 did not match in the multiple alignments (Fig. 3*C*). This raises the possibility that the difference between INMT-C168 and NNMT-A168 may be significant in determining the distinct activities of INMT and NNMT.

### INMT-L164 and E167, but not C168, are important for the methyltransferase activity

**Figure 4:**
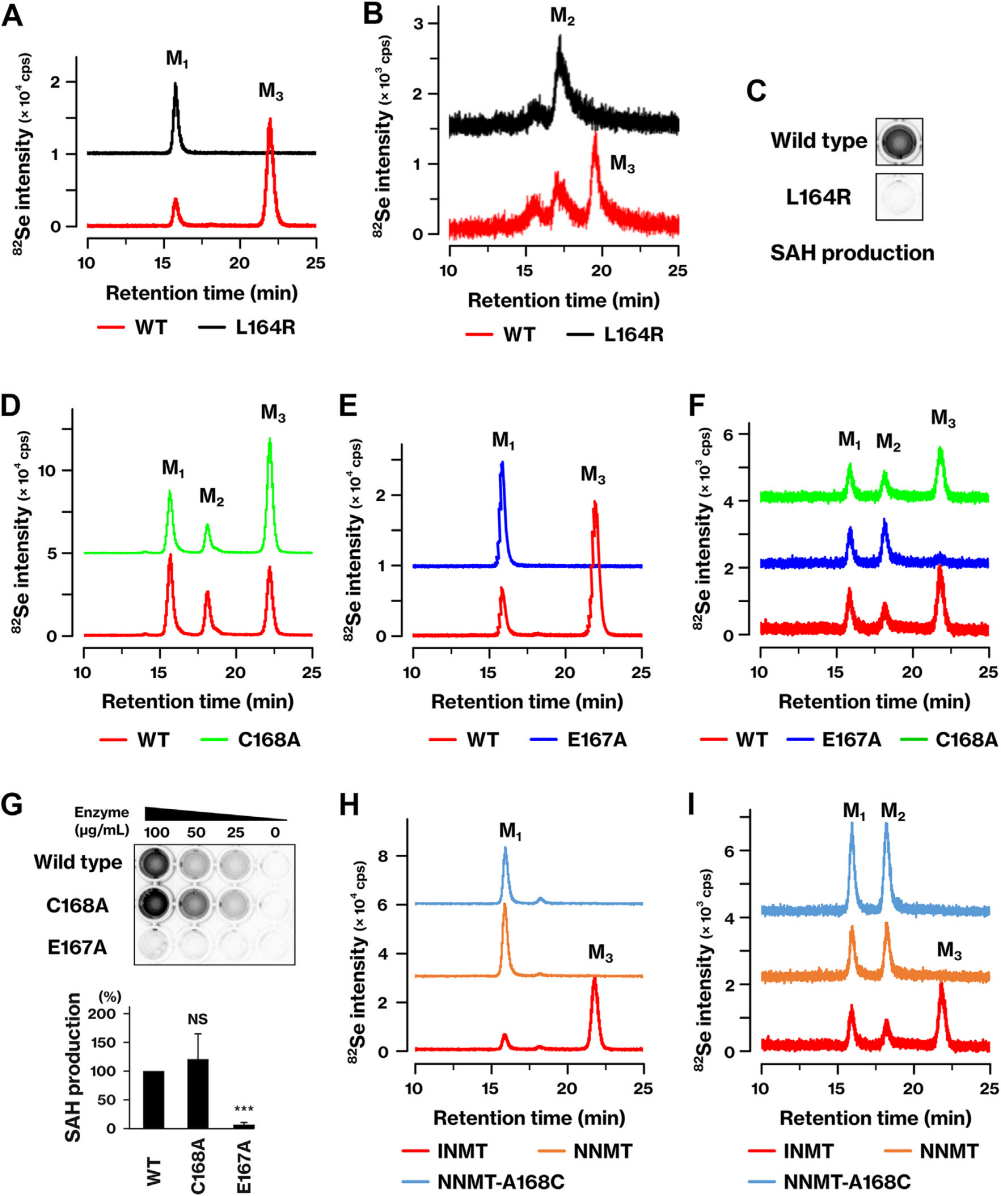
**Important role of INMT-L164 and E167, but not C168, in *Se*-methyltransferase activity.***A*, *B* and *D*‒*F*, the same experiments as those shown in Figure 1 were conducted. INMT-L164R, C168A, and E167A mutants were tested for their ability to methylate the monomethylated (*A*, *D* and *E*) or dimethylated form of Se (*B* and *F*). The reaction mixtures consisting of DMDSe (*A*, *D* and *E*) or DMSe (*B* and *F*), GSH, and methyltransferases were incubated, and the resulting reaction products were subsequently analyzed using LC‒ICP-MS. *C* and *G*, the *N*-methyltransferase activity of INMT mutants was evaluated using the MTase-Glo methyltransferase assay kit. The production of *S*-adenosylhomocysteine (SAH) was quantified by measuring chemiluminescence. Each well contained 100 μg/ml (*C*) or 0‒100 μg/ml (*G*) of INMT protein. The bar graph represents the results of three independent experiments. NS indicates no significant difference. ∗∗∗*p* < 0.001. *H* and *I*, the methyltransferase activity of the NNMT-A168C mutant was evaluated using the monomethylated (*H*) or dimethylated (*I*) form of Se. The results are representative of two (*C*, *H* and *I*) or more than three (*A*, *B* and *D*‒*F*) independent experiments. The data for the INMT WT in panels *F* and *I* were generated from the same experiment. M_1_, MSA. M_2_, DMSeO. M_3_, TMSe. cps, counts per second. GSH, glutathione; INMT, indolethylamine *N*-methyltransferase; NNMT, nicotinamide *N*-methyltransferase; TMSe, trimethylselenonium ion.

We investigated the role of the INMT-L164 and C168 residues in the methyltransferase activity of INMT. The INMT-L164R mutant showed a deficiency in the methylation of the mono- and di-methylated forms of Se (Fig. 4, *A* and *B*) and in the *N*-methylation of tryptamine (Fig. 4*C*). These results indicate that L164 is important for the methyltransferase activity of INMT. Considering the stable association of INMT-L164 with DMSe (Fig. 3, *D* and *F*), these findings suggest that INMT-L164 is one of the amino acid residues that stabilize the association of the methylation substrate in the active center.

On the basis of the results of the binding energy calculations and the multiple alignments shown in Figure 3, we generated the INMT-C168A and NNMT-A168C mutants. We also focused on E167 owing to its conservation in the INMT family. The pKa calculation performed on the H++ server (22) revealed that INMT-E167 has a pKa value of 7.25, indicating that the E167 residue in the active center is likely to be protonated. The INMT-C168A mutant exhibited proficiency in the methylation of the mono- and di-methylated forms of Se (Fig. 4, *D* and *F*) and in the *N*-methylation of tryptamine (Fig. 4*G*). Conversely, the NNMT-A168C mutant did not methylate the mono- or di-methylated form of Se (Fig. 4, *H* and *I*). In our preliminary experiments, the NNMT-A168C mutant demonstrated proficiency in methylating nicotinamide. These results indicate that the differential activity of INMT and NNMT is not a result of the disparity between INMT-C168 and NNMT-A168. The INMT-E167A mutant showed a deficiency in the methylation of the mono- and di-methylated forms of Se (Fig. 4, *E* and *F*) and in the *N-*methylation of tryptamine (Fig. 4*G*). These findings indicate that INMT-E167 is essential for the methyltransferase activity of INMT.

### INMT possesses a channel that connects the active center to the protein surface

**Figure 5:**
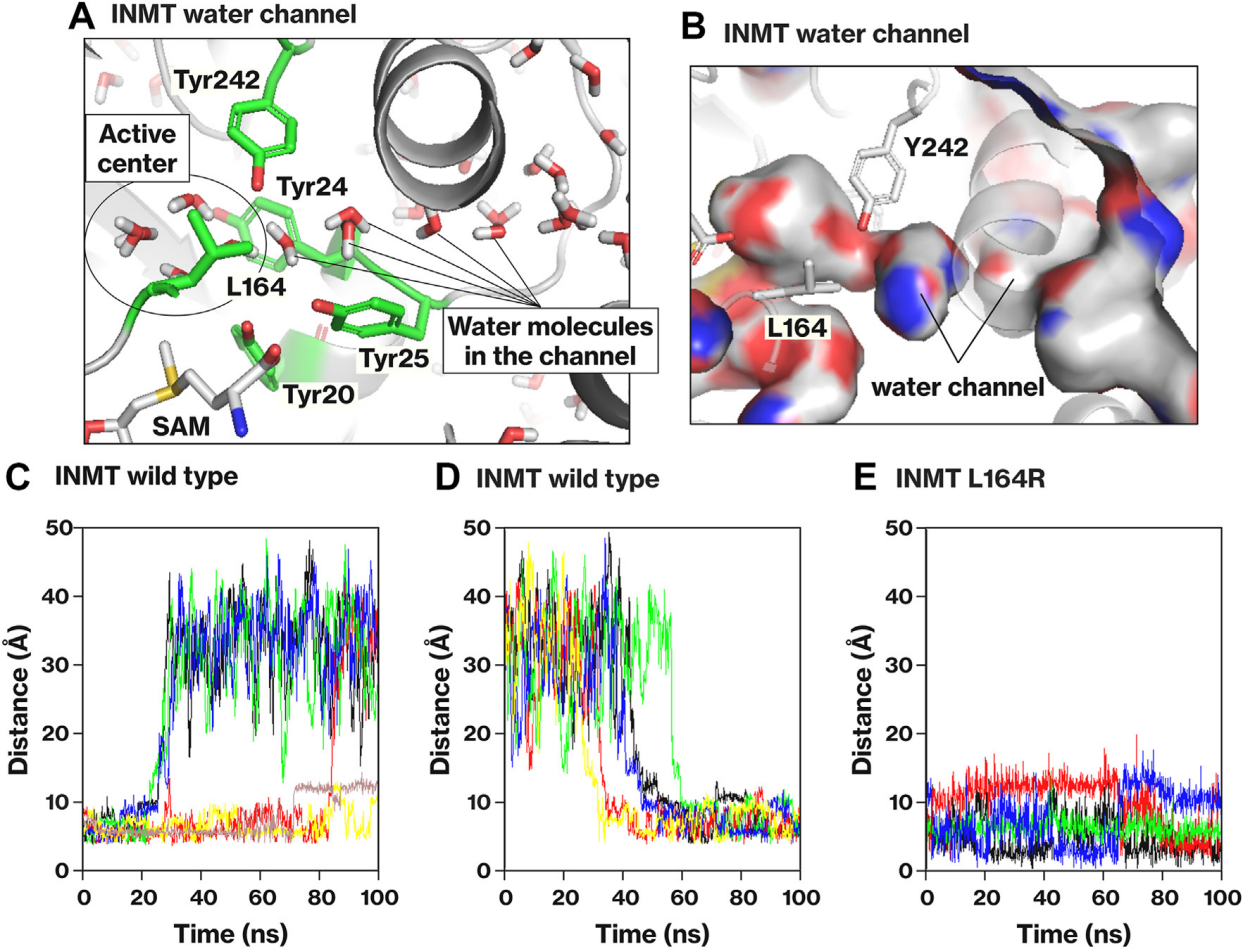
**Channel connecting the active center to the protein surface in INMT.***A* and *B*, the crystal structure of the INMT protein (PDB 2A14) was solvated with water molecules and then subjected to energy minimization using the steepest descent method. NVT and NPT equilibrations were conducted while restraining the positions of the INMT protein and SAM. The water molecules in the active center and the water channel are depicted in *panel A*, and the protein surface as seen from the inner side is illustrated in *panel B*. *C*–*E*, MD simulations were performed to analyze the dynamics of water molecules in the active center and the water channel of INMT. The simulations included INMT WT (*C* and *D*) and L164R (*E*) proteins. The distance between SAM and the water molecules was measured as a result. Energy minimization, equilibration, and MD simulations were performed using GROMACS. INMT, indolethylamine *N*-methyltransferase; MD, molecular dynamics; SAM, *S*-adenosylmethionine.

The active centers of the INMT family are embedded within the protein and shielded from the surrounding solvent (https://www.rcsb.org/structure/2A14) (12, 13). However, we observed the presence of a channel in INMT that establishes a connection between the active center and the protein surface (Fig. 5, *A* and *B*). During the MD simulation, it was observed that water molecules traversed through this channel (Fig. 5, *C* and *D*); water molecules initially occupied the active center but gradually diffused toward the protein surface. Meanwhile, additional water molecules entered the active center. Notably, the INMT-L164 residue was located near the junction of the active center and the water channel (Fig. 5, *A* and *B*). In the L164R mutant, water movement was impeded (Fig. 5*E*), suggesting that the L164 residue is located within the water channel. These findings suggest that INMT utilizes the channel to accommodate or release substrates.

### SNP rs886136672 (L164P) leads to the inactivation of INMT

**Figure 6:**
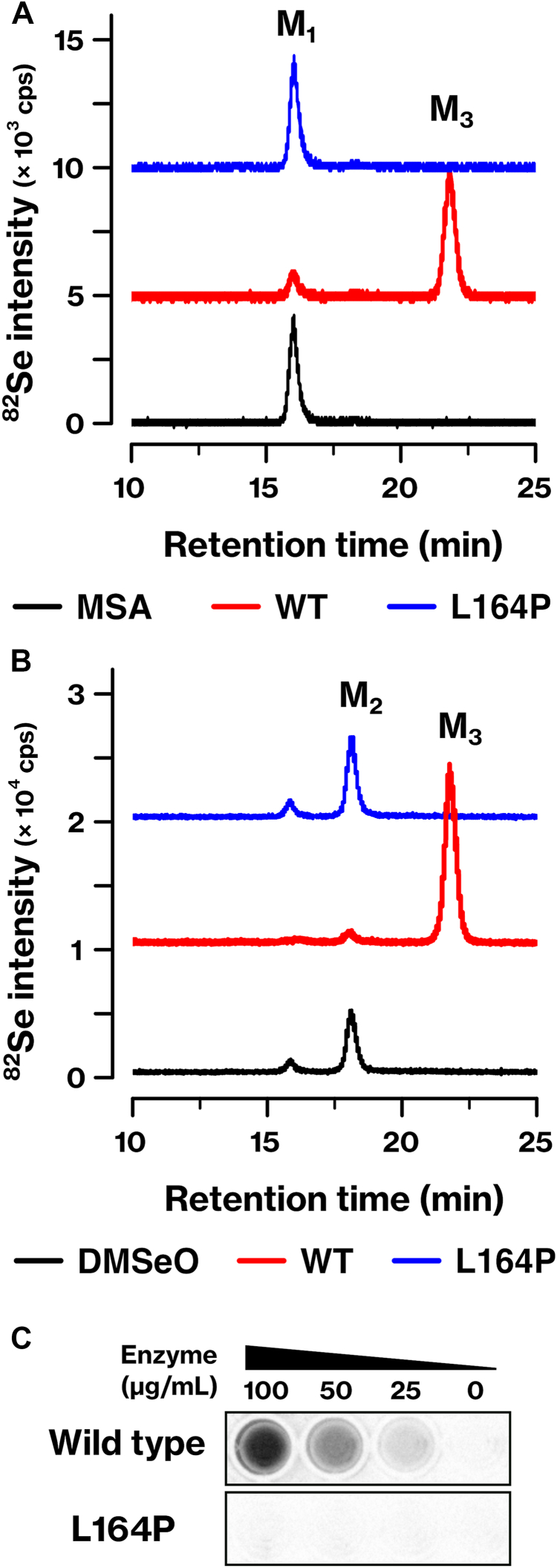
**Inactivation of INMT methyltransferase activity by SNP rs886136672.***A* and *B*, the methyltransferase activities of INMT WT and INMT-L164P mutant were assessed using the same experimental setup as that described in Figure 1. Monomethylated Se (*A*) and dimethylated Se (*B*) were used as substrates, and the reaction products were analyzed using LC‒ICP-MS. *C*, the *N*-methyltransferase activity of INMT-L164P mutant was evaluated using the MTase-Glo methyltransferase assay kit, following the same procedure as that depicted in Figure 4*G*. Representative results of three (*A*) or two (*B* and *C*) independent experiments are presented. M_1_, MSA. M_2_, DMSeO. M_3_, TMSe. cps, counts per second. INMT, indolethylamine *N*-methyltransferase; SNP, single nucleotide polymorphism; TMSe, trimethylselenonium ion.

We searched for single nucleotide polymorphisms (SNPs) that affect the active center of INMT. Specifically, SNP rs886136672 (T > C) introduces a missense mutation to yield INMT-L164P, which is found in approximately 0.01 to 0.03% of the African population (23). The INMT-L164P mutant exhibited a deficiency in the methylation of the mono- and di-methylated forms of Se (Fig. 6, *A* and *B*). In addition, *N*-methylation of tryptamine was impaired in the INMT-L164P mutant (Fig. 6*C*). These results indicate that SNP rs886136672 inactivates the methyltransferase activity of INMT.

### Quantitative analyses revealed the involvement of hydrophobic interactions through INMT-L164 in substrate binding and catalytic activity

We quantified the *V*_max_ and *K*_M_ values of INMT mutants to obtain quantitative information about their enzymatic activity. The INMT WT showed a *K*_M_ of 27.8 μM (Table 2 and Fig. S3), which may be sufficient for an enzyme involved in the *Se*-methylation reaction *in vivo*. By contrast, the L164R and L164P mutants showed no enzymatic activity. Additionally, we generated L164G and L164N mutants, and these mutants exhibited a decrease in both *V*_max_ and *K*_M_. The L164G mutation removed the large hydrophobic chains, while the L164N mutation introduced a positive charge in the active center. These results suggest that the hydrophobic interaction involving the L164 residue is crucial for both substrate binding and catalytic activity. These results align with the *in silico* analysis, which suggested the involvement of L164 in substrate binding (Fig. 3).

**Table 2:** Quantitative analyses of *Se*-methyltransferase activity of INMT

	*V*_max_ (nmol/min∙mg)	*K*_M_ (μM)
WT	22.7 ± 0.6	27.8 ± 1.5
L164G	7.2 ± 0.2	169.3 ± 2.1
L164N	8.0 ± 0.2	104.4 ± 2.1
L164R	0.3 ± 0.1	N.D.
L164P	0.3 ± 0.1	N.D.

The methylation reaction of DMDSe by INMT was analyzed quantitatively. The Michaelis–Menten equation was fitted to the observed data using nonlinear least squares to estimate the *V*_max_ and *K*_M_ values. The corresponding plots are shown in Fig. S3. The table represents results from more than three independent experiments. Values are shown as mean ± SD. The *K*_M_ values of L164R and L164P were unable to be determined. N.D., not determined.

### TPMT-R152 is important for the methyltransferase activity

**Figure 7:**
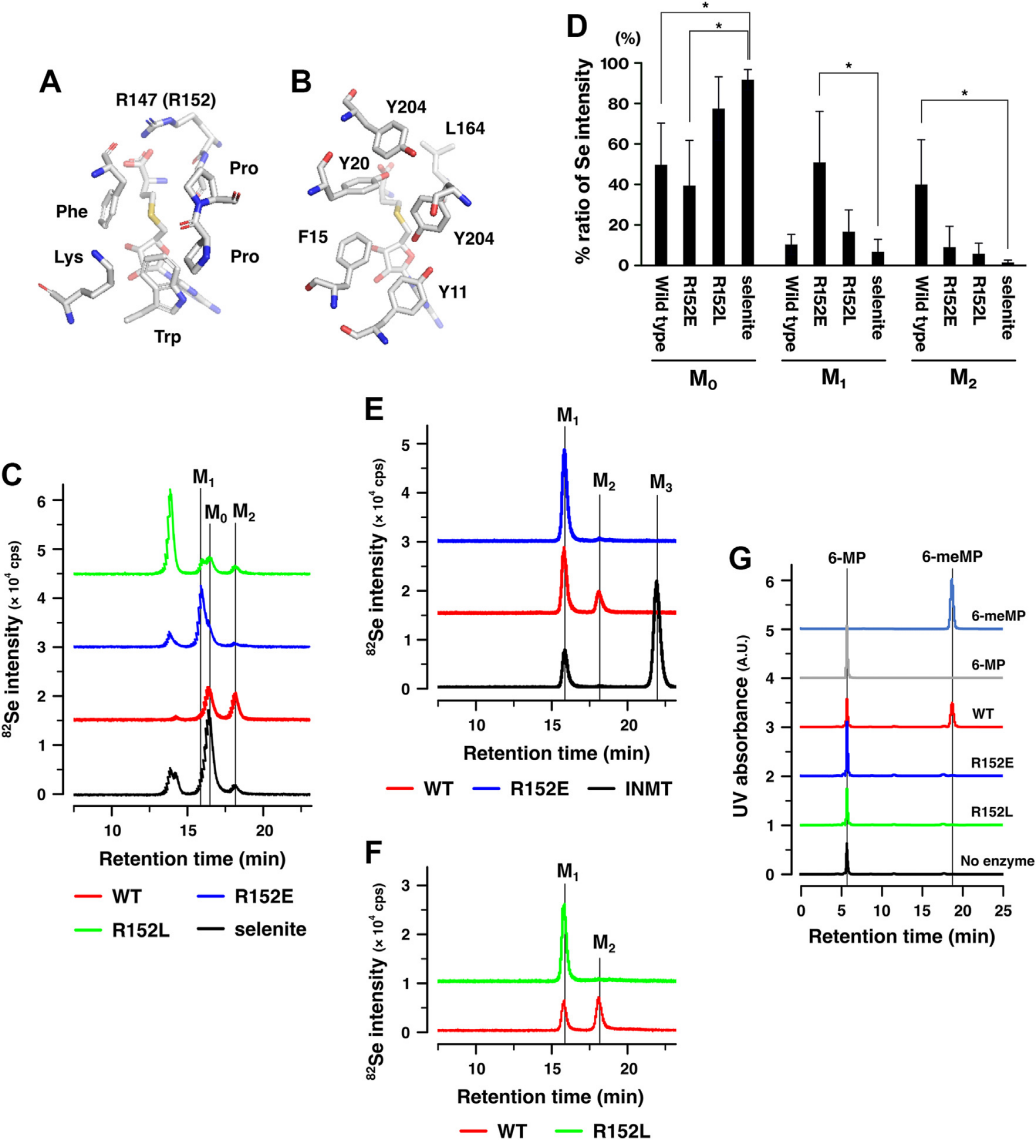
**TPMT-R152 plays a crucial role in *Se*- and *S*-methyltransferase activities.***A* and *B*, crystal structures of mouse TPMT (*A*, PDB 2GB4) and human INMT (*B*, PDB 2A14) are displayed, highlighting the amino acids present in their active centers. *C* and *D*, the methyltransferase activities of TPMT WT and R152E and R152L mutants were assessed using the non-methylated form of Se. Selenite, GSH, and human TPMT were incubated, and the reaction products were analyzed using LC‒ICP-MS. *Panel C* displays representative results of three independent experiments. The bar graph presents the ratios of Se compounds in the reaction products. Elution profiles were quantified using the Markov chain Monte Carlo method and the mixture distribution model. The percentages of the non-methylated form (M_0_) were calculated by adding the values of GSSeSG and selenite. *p*-values were calculated using either Student’s or Welch’s *t* test to determine the difference from selenite. ∗*p* < 0.05. *E* and *F*, the same experiments as in *Panel C* were conducted, except that the methylation of the monomethylated form of Se was examined. The reaction mixtures consisting of DMDSe, GSH, and human TPMT were incubated and analyzed using LC‒ICP-MS. The results are representative of more than two independent experiments. *G*, the *S*-methyltransferase activities of TPMT mutants were examined. The reaction mixtures, which consisted of recombinant proteins, SAM, and 6-mercaptopurine (6-MP), were incubated. The resulting reaction products were then analyzed using HPLC. *S*-methylated 6-mercaptopurine (6-meMP) and 6-MP were used as standards. Representative results of more than two independent experiments are shown. M_0_, selenite. M_1_, MSA. M_2_, DMSeO. M_3_, TMSe. cps, counts per second. GSH, glutathione; GSSeSG, selenodiglutathione; HPLC, high-performance liquid chromatography; INMT, indolethylamine *N*-methyltransferase; SAM, *S*-adenosylmethionine; TMSe, trimethylselenonium ion; TPMT, thiopurine *S*-methyltransferase.

We compared the structures of the active centers of TPMT and INMT. In the active centers, the R147 residue in mouse TPMT, corresponding to R152 in human TPMT, occupied the same position as the INMT-L164 residue (Fig. 7, *A* and *B*). A previous study has shown that the R152 mutant of TPMT is deficient in the methyltransferase activity for 6-MP (16). To investigate the role of R152 in the *Se*-methyltransferase activity of TPMT, we examined the methyltransferase activities of TPMT-R152E and R152L mutants. The TPMT-R152E mutant showed partial methyltransferase activity toward a nonmethylated form of Se (Fig. 7, *C* and *D*). A mono-methylated product, but not a di-methylated product, was generated with the TPMT-R152E mutant. In the case of the R152L mutant, the majority of Se remained in the nonmethylated form. Neither the TPMT-R152E mutant nor the R152L mutant methylated mono-methylated Se, indicating a deficiency in the second methylation step (Fig. 7, *E* and *F*). The TPMT-R152E mutant was incapable of methylating 6-MP (Fig. 7*G*), in line with previous findings (16). Similarly, the TPMT-R152L mutant also exhibited a deficiency in the methylation of 6-MP. These results demonstrate that the TPMT-R152 mutants were deficient in *S*-methyltransferase activity.

### The methyltransferase activities of TPMT and INMT are not interchangeable

We investigated whether the *Se*-methyltransferase activity of TPMT and INMT could be interchanged and found that the TPMT-R152E and R152L mutants were unable to convert monomethylated Se into TMSe (Fig. 7, *E* and *F*) nor were they able to methylate DMSe (data not shown). Likewise, the INMT-L164R mutant, the INMT-L164R/E166L mutant, and the INMT-L164R/E166R mutant were unable to methylate the nonmethylated form of Se (data not shown). These results indicate that the *Se-*methyltransferase activities of TPMT and INMT are not interchangeable.

## Discussion

In our previous study, we demonstrated that the mono- and di-methylation of Se are facilitated by TPMT, whereas the di- and tri-methylation are catalyzed by INMT. Here, we investigated the *Se*-methyltransferase activity of the INMT family and found that only INMT exhibited *Se*-methyltransferase activity. By comparing substrate binding, we identified crucial amino acid residues in INMT that are involved in substrate recognition. In addition, through a comparison of these key residues with those in TPMT, we uncovered distinct molecular mechanisms underlying substrate recognition by INMT and TPMT.

In the INMT family, only INMT demonstrated the ability to methylate Se compounds (Fig. 1). Even though INMT and NNMT utilized the same amino acid residues for substrate recognition, there was a disparity in the balance of binding energy between them (Fig. 3). The distinct activity of each methyltransferase could be attributed to the spatial organization of key amino acid residues. The difference between INMT-C168 and NNMT-A168 did not affect their methyltransferase activities (Fig. 4, *D*, *F*‒*I*). Although INMT-C168 was conserved in the alignment, its function remained unclear. On the other hand, INMT-E167 was essential for the *Se*- and *N*-methyltransferase activities of INMT (Fig. 4, *E*‒*G*). The INMT-E167 is conserved as Asp in NNMT and as Glu in PNMT. The corresponding mutation, PNMT-E185 substitution, also affected the methyltransferase activity (12). The precise molecular function of these Glu and Asp residues has yet to be determined. Previous research has suggested that substrate deprotonation may be one of the functions of PNMT-E185 (12). However, substrate deprotonation is not necessary for the methylation of DMSe by INMT. Another potential role of these residues is to stabilize the methyl cation during the transition states in the methylation reaction. INMT is highly expressed in the lungs (7, 10). Given that NNMT and PNMT did not mediate the methylation of Se (Fig. 1), the current data support our previous suggestion that TMSe is produced by INMT in the lungs (7).

The active center of INMT is embedded within the protein and surrounded by hydrophobic residues (Figs. 5*A* and 7*A*). In contrast, the active center of TPMT is exposed to the solvent and consists of basic and hydrophobic residues (Fig. 7*A*) (16). INMT has a water channel that facilitates the inclusion of substrates or the release of products (Fig. 5). This channel was obstructed in the L164R mutant (Fig. 5), suggesting that INMT-L164 may capture substrates passing through it. Indeed, the results obtained with the INMT-L164G and L164N mutants indicate that L164 is involved in substrate binding through hydrophobic interactions (Table 2 and Fig. S3). The hydrophobic residues in the INMT active center play a crucial role in the *Se*- and *N*-methylation reactions (Figs. 3, 4 and 6). These findings suggest that INMT recognizes electrically neutral substrates through the hydrophobic residues in its inner space active center. On the other hand, R152 was found to be essential for the *Se*-methyltransferase activity of TPMT (Fig. 7). TPMT-R152 was also found to be crucial for *S*-methylation (Fig. 7), as previously reported (16). These findings indicate that TPMT recognizes negatively charged substrates using the active center located on the protein surface. The selenol group and H_2_Se have pKa values of 5.4 and 3.9, respectively, suggesting that TPMT is capable of recognizing HSe^−^, glutathione selenopersulfide (GSSe^−^), and the deprotonated form of methaneselenol (CH_3_Se^−^). In contrast, the substrates of INMT include DMSe and the protonated form of methaneselenol (CH_3_-SeH). These results suggest that INMT and TPMT recognize substrates on the basis of their charges, thereby explaining why the methylation of Se requires two different methyltransferases. The first and second methylation steps involve negatively charged substrates, whereas the second and third methylation steps involve electrically neutral substrates.

Methaneselenol underwent methylation by INMT (Fig. 1*A*). Within the active center of INMT, the selenol group is expected to have a high pKa value in the hydrophobic environment and to become protonated. Indeed, analysis conducted using the H++ server (22) predicted a pKa value of 7.25 for INMT-E167. Therefore, we propose that INMT is likely to recognize methaneselenol in its protonated form as a neutral molecule. On the other hand, TPMT recognizes methaneselenol in its deprotonated form owing to the solvent-exposed nature of its active center (16). It is common for many methyltransferases to promote substrate deprotonation, which facilitates the nucleophilic attack of substrates on the methyl group in SAM. If our proposal is correct, the methylation of methaneselenol by INMT is a unique exception that requires the substrates to be protonated in order for the methylation reaction to occur.

TPMT and INMT share a common substrate in the form of mono-methylated Se, which raises the possibility that these two enzymes may have a similar substrate recognition mechanism. However, our examination of the mutated proteins revealed that the activities of INMT and TPMT were not interchangeable (Fig. 7, *E* and *F* and data not shown). Specifically, the INMT-L164R mutant did not methylate the nonmethylated forms of Se, and the TPMT-R152L mutant did not produce TMSe. It is likely that not only the residues involved in substrate recognition but also the hydrophobic and basic environments of the active center are necessary for substrate recognition.

The TPMT-R152E mutant was found to be deficient in the methylation of the monomethylated form of Se but proficient in the methylation of the nonmethylated form of Se (Fig. 7). TPMT is responsible for methylating selenocysteine to produce *Se*-methyl selenocysteine (17). The TPMT-R152E mutant may recognize the structure of cysteine in GSSe^−^ by using the same residues as those used for the recognition of selenocysteine. In the methylation of the monomethylated form of Se, the substrate should be the deprotonated form of methaneselenol (CH_3_Se^−^), and the positive charge on the R152 residue should play a crucial role. Indeed, previous research has disclosed an interaction between R152 and the sulfur of 6-MP during the methylation of 6-MP (16).

A genome-wide association study revealed SNPs in *INMT* that affect the amount of TMSe in urine (24). In this case, individuals homozygous for the major allele, heterozygotes, and individuals homozygous for the minor allele showed different amounts of TMSe in urine, indicating that both homozygous and heterozygous mutations affect the metabolism involving INMT activity. Previous work examined the effect of SNPs in the coding region of *INMT* on the methyltransferase activity of recombinant INMT protein. The combination of SNPs rs2302340 and rs2302339 resulted in INMT variants V205/E219 and M205/G219 having similar K_m_ values (11). The alternate alleles of rs2302340 and rs2302339 occurred at frequencies of 41% and 31%, respectively (23). SNP rs4720015 resulted in F254 and C254 variations. These mutants differed in the *N*- and *S*-methyltransferase activities but retained the *N*-, *S*-, and *Se*-methyltransferase activities (25). The minor allele occurred at a frequency of 11% (23). Unlike these SNPs, SNPs that affect INMT activity were much less frequent. SNP rs77743549 resulted in the INMT-H46P mutant that was deficient in *Se*-methyltransferase activity (25) and occurred at a frequency of 0.8% (23). This mutation occurred outside the active center and might affect protein folding. Some SNPs resulted in mutations within the active center and affected the amino acid residues that were found to be involved in substrate recognition (Fig. 3*A*). SNPs rs182818909, rs886136672, and rs1284904998 resulted in Y24 C, L164P, and Y204C mutations, respectively. However, their frequencies were less than 0.1%. INMT-L164P abolished both *Se*- and *N-*methyltransferase activities (Fig. 6). By contrast, the polymorphisms of TPMT, including those that inhibit the methyltransferase activity, are well known, and 0.3% of Caucasians have been reported to have no detectable TPMT activity (26, 27). These observations indicate that the methyltransferase activity of INMT is essential for certain aspects of homeostasis.

INMT exhibits *N*-, *S*-, and *Se*-methyltransferase activities. However, it is still unclear whether all of these activities are necessary or if one or two activities may be dispensable. Recent research has shown that the recombinant rat INMT protein does not possess *N*-methyltransferase activity toward tryptamine. It was also reported that tissue homogenates did not exhibit any activity in producing *N,N*-dimethyl- and *N*-methyltryptamine (28). In rats, the excretion of Se as TMSe in urine has been well documented (5). One possibility is that the rat INMT protein exclusively retains *Se*- and *S*-methyltransferase activities. If this is the case, it presents an intriguing scenario where the *Se*- and *N*-methyltransferase activities of INMT have diverged, suggesting that INMT is conserved for the metabolism of Se and S. Another possibility is that other methyltransferases facilitate the production of TMSe in rats.

INMT has two paralogs, NNMT and PNMT, but only INMT exhibits *Se*-methyltransferase activity, which reinforces its role in the urinary excretion of Se. Multiple alignments of the INMT family indicate that INMT is conserved in mammals, birds, reptiles, and amphibians (Figs. 3*C* and S2, and data not shown). TMSe production has been observed in mammals, birds (29), and reptiles (30). The toxicity of DMSe is significantly lower than that of selenide and methaneselenol. This suggests that methylation by TPMT is sufficient for detoxifying Se and eliminating volatile DMSe through breathing. However, mammals and other organisms have evolved to convert DMSe into TMSe using INMT and to excrete TMSe in urine. The significance of the third methylation step is still uncertain. However, the low frequency of SNPs in the substrate-binding residues of INMT indicates the crucial role of the third methylation step.

Our findings suggest that TPMT recognizes negatively charged substrates, whereas INMT recognizes electrically neutral substrates using a hydrophobic active center that is embedded within the protein. These findings clarify the necessity for the sequential action of the two methyltransferases in the production of TMSe. The conservation and frequency of SNPs in *INMT* suggest that the residues responsible for substrate recognition are highly conserved, whereas other residues exhibit variability. This underscores the physiological importance of TMSe production in the human body. Further research will provide insights into the role of TMSe production in maintaining homeostasis.

## Experimental procedures

### Purification of recombinant proteins

ORFs of INMT, NNMT, and PNMT were synthesized by Twist Biosciences and cloned into pET29b vectors, which included C-terminal histidine tags. The recombinant proteins were expressed in BL21(DE3) cells and purified following the previously described protocol (31). Human TPMT was expressed from the pBAD vector in Top10 bacteria, as previously described (7). In brief, the recombinant protein expression was induced using 0.5 mM IPTG or 0.2% arabinose at 37 °C for 3‒4 h. The cells were collected by centrifugation and suspended in a buffer containing 20 mM NaPi (pH 6.8), 300 mM NaCl, 0.5 mM PMSF, 2 μg/ml aprotinin, 0.8 μg/ml pepstatin A, 2 μg/ml leupeptin, and 0.5 mM EGTA. The cells were digested with 1 mg/ml of lysozyme for 15 min on ice and then lysed with three cycles of freeze and thaw. Genomic DNA was fragmented by sonication, and the resulting lysate was supplemented with 0.1% Triton X-100 and incubated on ice for 15 min. The lysate was cleared through centrifugation, and the resulting supernatant was collected.

The lysate was mixed with TALON metal affinity resin (635502, TaKaRa) for 1‒2 h in a cold room. The resin was washed seven times using a wash buffer that contained 20 mM NaPi (pH 6.8), 300 mM NaCl, and 0.1% Triton X-100. The bound proteins were eluted using the wash buffer that contained 300 mM imidazole. The elution fraction was dialyzed overnight in a cold room against a dialysis buffer consisting of 20 mM Tris–HCl (pH 7.5), 100 mM NaCl, 50% glycerol, and 0.1% Triton X-100 using a Slide-A-Lyzer Dialysis Cassette with a 10 kDa molecular weight cut off (Cat. No. 66380, Thermo Fisher Scientific). After purification, we confirmed the *N*-methyltransferase activity of NNMT and PNMT on nicotinamide and noradrenaline, respectively, by utilizing the MTase-Glo methyltransferase assay kit (V7601, Promega). The activity of NNMT was also confirmed by monitoring the methylation of nicotinamide to 1-methyl nicotinamide using HPLC coupled with a UV detector (data not shown).

### *Se-*methylation reaction and LC‒ICP-MS

The *Se*-methyltransferase activity was examined using the procedure described in a previous study (7). The reaction mixture comprised 1 mM SAM, 10 mM GSH, 20 mM sodium phosphate buffer (pH 7.4), and 100 μg/ml of recombinant methyltransferases. The substrates used were 10 μM of selenite, dimethyldiselenide, or DMSe. The reaction proceeded at 37 °C for 4 h. The reaction was stopped by incubating it with 3% hydrogen peroxide for 30 min. Volatile methaneselenol and dimethylselenide were oxidized to methaneseleninic acid and dimethylselenoxide, respectively. Excess hydrogen peroxide was degraded by treating the mixture with 0.1 μg/ml of catalase at 37 °C for 1 h. The mixture was filtered through a 0.45 μm filter (#SLLHH04NL, Merck Millipore), and the resulting filtrate was used for analysis.

The *Se*-methylation reaction was analyzed using a hyphenated LC‒ICP-MS technique. The HPLC system consisted of an HPLC pump (GL-7410, GL Science) and a multimode size-exclusion column (Shodex Asahipak GS-320HQ, 7.5 mm i.d. × 300 mm, 6 μm, Resonac). The injection volume was set to 20 μl. The column was eluted with 50 mM ammonium acetate (pH 6.5) at a flow rate of 1.0 ml/min. The eluate was introduced directly into the nebulizer of the ICP-MS (Agilent 8800 ICP-QQQ, Agilent Technologies), and Se was detected at *m*/*z* 82. Standards of Se compounds were prepared as previously described (7), and elution profiles of the standard compounds were obtained (Fig. S2). Table S1 presents a list of the Se compounds that were added to the reaction, their probable forms during the reaction, and the chemical forms that were analyzed using LC‒ICP-MS.

### *S-*methylation reaction of TPMT

The *S*-methyltransferase activity of TPMT was examined as described previously (7). In brief, the reaction mixture was composed of 0.5 mM 6-MP, 1 mM SAM, 20 mM sodium phosphate (pH 7.4), and 100 μg/ml TPMT protein. The reaction mixture was analyzed using HPLC with a Symphonia C18 column (4.6 mm i.d. × 150 mm, 5 μm, JASCO) and a buffer consisting of 37.5 mM ammonium phosphate (pH3.4) and 20% methanol, at a flow rate of 0.5 ml/min. The HPLC system was comprised of an HPLC Pump (PU-1580, JASCO) with a ternary gradient unit (LG-1580-02, JASCO), a degasser (DG-4580, JASCO), a column oven (CO-1565, JASCO), and a UV-VIS detector (UV-4075, JASCO). Data was acquired by a system controller LC-NetII/ADC and ChromeNAV (JASCO).

### *N-*methylation reactions of INMT, NNMT, and PNMT

The *N*-methyltransferase activity of INMT, NNMT, and PNMT was examined using tryptamine, nicotinamide, and noradrenaline as substrates. The reaction was monitored using the MTase-Glo methyltransferase assay (V7601, Promega) in accordance with the manufacturer’s instructions. In this assay, methylation reactions were traced by detecting the production of *S*-adenosylhomocysteines, which was quantified using chemiluminescence. The reaction mixture comprised 20 mM Tris–HCl (pH 8.0), 50 mM NaCl, 3 mM MgCl_2_, 1 mM EDTA, 1 mM DTT, 0.1 mg/ml bovine serum albumin, 100 μM SAM, and the MTase-Glo reagent. Substrates used were 10 μM tryptamine, 10 μM nicotinamide, and 10 μM L-noradrenaline. The recombinant protein was added to the reaction mixture at a concentration of 25‒100 μg/ml and incubated for 30 min at room temperature. After the reaction, the MTase-Glo detection solution was added, and the mixture was incubated for an additional 30 min. Chemiluminescence was detected and quantified using the ChemiDoc XRS+ (Bio-Rad).

The *N*-methylation reaction of NNMT was also analyzed using HPLC, following the method described by others (32, 33). The reaction mixture comprised 20 μM nicotinamide, 9 μM SAM, 2 mM DTT, 100 mM Tris-HCl (pH 7.5), 0.04% bovine serum albumin, and 1 mg/ml NNMT. The reaction products were analyzed using HPLC with a C18 column and a buffer consisting of 7 mM sodium 1-heptane sulphonic acid, 5 mM potassium dihydrogen phosphate, 20 mM trimethylamine, and 0.1 mM ascorbic acid. Elution was monitored on a UV detector, and nicotinamide and 1-methyl nicotinamide were used as standards.

### A Bayesian method for quantitative analysis of LC‒ICP-MS

We applied a Bayesian statistical approach to quantify the LC‒ICP-MS data presented in Figure 7*C*. The elution profiles were converted into one-dimensional probability distributions, and the resulting distributions were modeled using probability density functions. The parameters of these distributions were estimated using PyMC3 v3.11.4 *via* Markov chain Monte Carlo sampling (34). The No-U-Turn Sampler (NUTS) in PyMC3 was utilized to sample from the posterior distribution.

To model the elution profiles of the standard compounds GSSeSG, methaneseleninic acid, selenite, and dimethylselenoxide, we utilized an exponentially modified Gaussian distribution (EMG). The EMG is defined as follows:

$$EMG\left(x;\mu ,\sigma ,\lambda \right)=\frac{\lambda }{2}\exp\left(\frac{\lambda }{2}\left(2\mu +\lambda \sigma^{2}-2x\right)\right)\cdot erfc\left(\frac{\mu +\lambda \sigma^{2}-x}{\sqrt{2}\sigma }\right)$$

Here, “exp” represents the exponential function, and “erfc” represents the complementary error function. We assigned continuous uniform distributions as the prior probabilities for the parameters *μ*, *σ*, and *λ* and estimated them for each standard compound using NUTS. The determined parameters, $\mu_{k}$, $\sigma_{k}$, and $\lambda_{k}$, were utilized to construct the EMG distributions for each compound in the subsequent development of the mixture model.

We modeled the elution profile of reaction mixtures using a mixture model consisting of multinomial and EMG distributions.

$$p\left(z|\omega \right)=\prod_{k=1}^{4}\omega_{k}^{z_{k}}$$

$$p\left(x|z,\omega \right)=\prod_{k=1}^{4}{\left\{EMG\left(x;\mu_{k},\sigma_{k},\lambda_{k}\right)\right\}}^{z_{k}}$$

The prior for the mixture weight ***ω*** was defined using a Dirichlet distribution (Dir) with ***α*** = (1, 1, 1, 1).

$$p\left(\omega |a\right)=Dir_{k=4}\left(\omega ;a\right)$$

The likelihood was defined as a mixture model of EMG distributions:

$$p\left(x|\omega \right)=\sum_{k=1}^{4}\omega_{k}EMG\left(x;\mu_{k},\sigma_{k},\lambda_{k}\right)$$

The weight of the mixture components, denoted as *ω*, which represents the proportion of Se compounds in the reaction mixture, was estimated for each reaction through posterior sampling using NUTS algorithm. The ratio of the nonmethylated form (M_0_) was calculated by adding the values of GSSeSG and selenite. The results were presented as the mean ± SD. The significance of the difference between selenite and the other compounds was analyzed using either Student’s or Welch’s *t* test, and *p*-values were calculated.

### MD simulation

The structures of human INMT (PDB 2A14), mouse NNMT (PDB 5YJI) (13), and human PNMT (PDB 3HCB) (12) were obtained from the Protein Data Bank. Protonation states were assigned using the H++ server (22). The topology of the methyltransferases was generated by tLEaP using the Amber ff19SB force fields (35). *S*-adenosylhomocysteines, present in the methyltransferase structure, were modified to SAM using Avogadro 1.95.1 (36). The topology of SAM was generated with ANTECHAMBER in AmberTools21 (37) and AM1-BCC (38) using the general AMBER force field 2 (GAFF2) (39). Since GAFF2 lacks parameters for Se, DMS was placed in the active center of methyltransferases. The methyltransferase, SAM, and DMS were then positioned in a truncated octahedral box with periodic boundaries set at a minimum distance of 12 Å from the solute to the box edge. The simulation box was filled with OPC water and by adding Na^+^ or Cl^−^ ions were added to neutralize the system. The topology and coordinate files were converted to GROMACS format using ParmEd (40).

The topology of DMSe was generated by modifying that of DMS. The topology of DMS was created with ANTECHAMBER and tLEaP using GAFF2 (39) and then converted to GROMACS format using ParmEd (40). Restrained electrostatic potential charge (41) of DMSe was generated with GAMESS 2018.R3 (42) and PyRED server (43, 44). Specifically, the geometry of DMSe was optimized using Hartree-Fock theory in combination with a 6-31G(d) basis set, referred to as HF/6-31G(d), with gradient convergence tolerance of 1.0 × 10^−5^ Hartree/Bohr. Subsequently, the electrostatic potential was calculated using HF/6-31G(d). The electrostatic potential was then converted to restrained electrostatic potential charge using the PyRED server. The Lennard-Jones parameters of Se were set to σ = 0.377741 nm and ε = 1.21754 kJ/mol in the GROMACS topology file (45, 46, 47). Other parameters were also modified to replace the sulfur with Se.

MD simulation was performed using GROMACS 2020.6 (48) compiled with CUDA 11.0. The structure was energy-minimized using the steepest descent method with a threshold of 1000 kJ/mol. A leapfrog integration scheme was applied with a time step of 2 femtoseconds. Bond lengths were constrained using the LINCS method (49). The Verlet list was used as the nonbonded cut-off scheme, with a cut-off of 12 Å for van der Waals and short-range electrostatic interactions. Long-range electrostatic interaction was calculated with the particle-mesh Ewald method (50). A three-dimensional periodic boundary condition was applied. The temperature and pressure were maintained at 310 K and 100 kPa, respectively. Position restraints were applied to the heavy atoms of methyltransferase, SAM, and DMSe, and NVT and NPT equilibrations were performed for 100 picoseconds each. Subsequently, the position restraints were released, and the production MD run was performed for data collection for 100 nanoseconds. After the run, the trajectory was converted with the TRJCONV module. The DISTANCE module was utilized to calculate the distance between the sulfur of SAM and Se of DMSe, as well as the distance between DMSe and amino acid residues.

### Fragment molecular orbital method

The interaction energy between DMSe and each residue of the methyltransferases was calculated using the FMO method (20, 21) with GAMESS 2021 R.2 (42, 51). The polypeptide of the methyltransferase was divided into fragments, with each fragment containing one amino acid residue (20, 21). The molecular orbitals of the fragments were calculated using the third generation of density functional tight binding (DFTB3) method with the 3OB-3-1 parameter set (52, 53), considering the water phase and employing the conductor-like polarizable continuum model (PCM), as described previously (54). As the 3OB-3-1 parameter set lacks parameters for Se, sulfur was used as a replacement. Electron dispersion was accounted for by implementing Grimme’s DFT dispersion correction with Becke-Johnson damping, known as DFT-D3(BJ) (55). The final free energy, including dispersion correction, was calculated using the FMO method with either two-body (FMO2) or three-body expansion (FMO3).

The averaged structure was made from the MD simulation trajectory using the RMSF module of GROMACS, and the geometry was optimized using the steepest descent method with GROMACS. Input files for GAMESS were prepared using Facio 23.1.5 (56). The structure was further optimized through Hessian update with FMO2-DFTB3/PCM<1> for 20 steps. The final free energy was calculated for methyltransferase/SAM/DMSe (*E*_*complex*_), methyltransferase/SAM (*E*_*dimer*_), and DMSe (*E*_*DMSe*_) using FMO3-DFTB3/PCM<1>. The binding energy was defined as *ΔE*_*bind*_ = *E*_*complex*_ − (*E*_*dimer*_ + *E*_*DMSe*_) (21). The interaction energy between DMSe and each amino acid residue was calculated using FMO2-DFTB3/PCM<1>, and the interactions were analyzed using pair interaction energy decomposition analysis (57).

### Quantitative analyses of *Se*-methylation reactions

The *V*_max_ and *K*_M_ values were measured using the MTase-Glo methyltransferase assay kit. The reaction mixture for *Se*-methylation comprised 50 μM SAM, 5 mM GSH, 20 mM sodium phosphate buffer (pH 6.8), 0.01% Triton X-100, and 5 μg/ml of recombinant methyltransferases. Dimethyldiselenide was used as the substrate at concentrations of 0 μM, 10 μM, 100 μM, 250 μM, 500 μM, 1.0 mM, 1.5 mM, and 2.5 mM. The reaction proceeded at 37 °C for 30 min and was terminated by adding 0.1% TFA. The *S*-adenosylhomocysteine generated in the reaction was quantified using the MTase-Glo kit, following the manufacturer’s instructions. Chemiluminescence was quantified using the SpectraMax iD3 (Molecular Devices). The Michaelis–Menten equation was fitted to the observed data through nonlinear least square analysis to estimate the *V*_max_ and *K*_M_. The *K*_M_ and *V*_max_ were defined using a Gaussian distribution.

$$p\left(K_{M}|\mu_{K},\lambda_{K}\right)=Norm\left(K_{M};\;\mu_{K},\lambda_{K}\right)$$

$$p\left(V_{\max}|\mu_{V},\lambda_{V}\right)=Norm\left(V_{\max};\;\mu_{V},\lambda_{V}\right)$$

The initial values for the means, $\mu_{K}$ and $\mu_{V}$, were estimated using gradient descent with the minimize function from the optimize module in SciPy v1.7.3. The loss function was defined using the Michaelis–Menten equation, incorporating the initial reaction rate $v_{0}$ and the initial substrate concentration $S_{0}$.

$$Loss=\sum_{i}{\left(v_{0i}\;-\;\frac{V_{\max}{\;S}_{0i}}{K_{M}+S_{0i}}\right)}^{2}$$

The prior distributions for the precisions, $\lambda_{K}$ and $\lambda_{V}$, were specified using a gamma distribution. The likelihood was modeled using a Gaussian distribution.

$$p\left(v_{0}|v_{K_{M},V_{\max}},\varepsilon \right)=Norm\left(v_{0};v_{K_{M},V_{\max}},\varepsilon \right)$$

$v_{K_{M},V_{\max}}$ was calculated using the Michaelis–Menten equation with the parameters *K*_M_, *V*_max_, and $S_{0}$. The precision ε was also defined using a gamma distribution. The parameters were estimated using PyMC3 through Markov chain Monte Carlo sampling. The NUTS algorithm was used to sample from the posterior distribution. The results were visualized using Matplotlib v3.5.2.

## Data availability

All data are contained within the manuscript.

## Supporting information

This article contains supporting information.

## Conflict of interest

The authors declare that they have no conflicts of interest with the contents of this article.
