# Supplementary material for: Differential molecular mechanisms of substrate recognition by selenium methyltransferases, INMT and TPMT, in selenium detoxification and excretion
Source: J Biol Chem. 2023 Dec 28;300(2):105599. doi: 10.1016/j.jbc.2023.105599 (PMC10844679; doi:10.1016/j.jbc.2023.105599)
Supplement: Supporting Table S1 and Figures S1–S3 [file mmc1.pdf]

Table S1. List of Se compounds used in the methylation reaction.

	chemical added to the reaction	probable forms in the reaction	chemical form analyzed by LC–ICP-MS
M_0, non-methylated	selenite	H_2Se, GS-SeH	selenite
M_1, monomethylated	dimethyldiselenide (DMDSe)	CH_3-SeH, GS-Se-CH_3	methaneseleninic acid (MSA)
M_2, dimethylated	dimethylselenide (DMSe)	CH_3-Se-CH_3	dimethylselenoxide (DMSeO)
M_3, trimethylated	———	———	trimethylselenonium ion (TMSe)

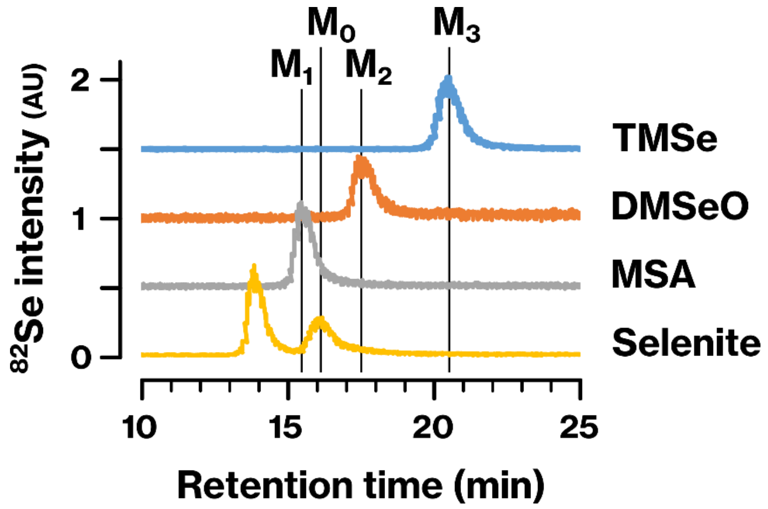

Figure S1. Elution profiles of standard compounds.

Standard compounds for *Se*-methylation reactions were analyzed using LC–ICP-MS. The elution profiles are presented. Selenite was eluted at 16.2 minutes. When selenite was mixed with GSH, another peak appeared at 13.8 minutes, which corresponds to GSSeSG. Dimethylselenide and dimethyldiselenide underwent oxidation using hydrogen peroxide and were injected as dimethylselenoxide (DMSeO) and methaneseleninic acid (MSA), respectively. The vertical axis was adjusted to accommodate all elution profiles within the same panel. TMSe, trimethylselenonium ion. M_0, selenite. M_1, MSA. M_2, DMSeO. M_3, TMSe. cps, counts per second.

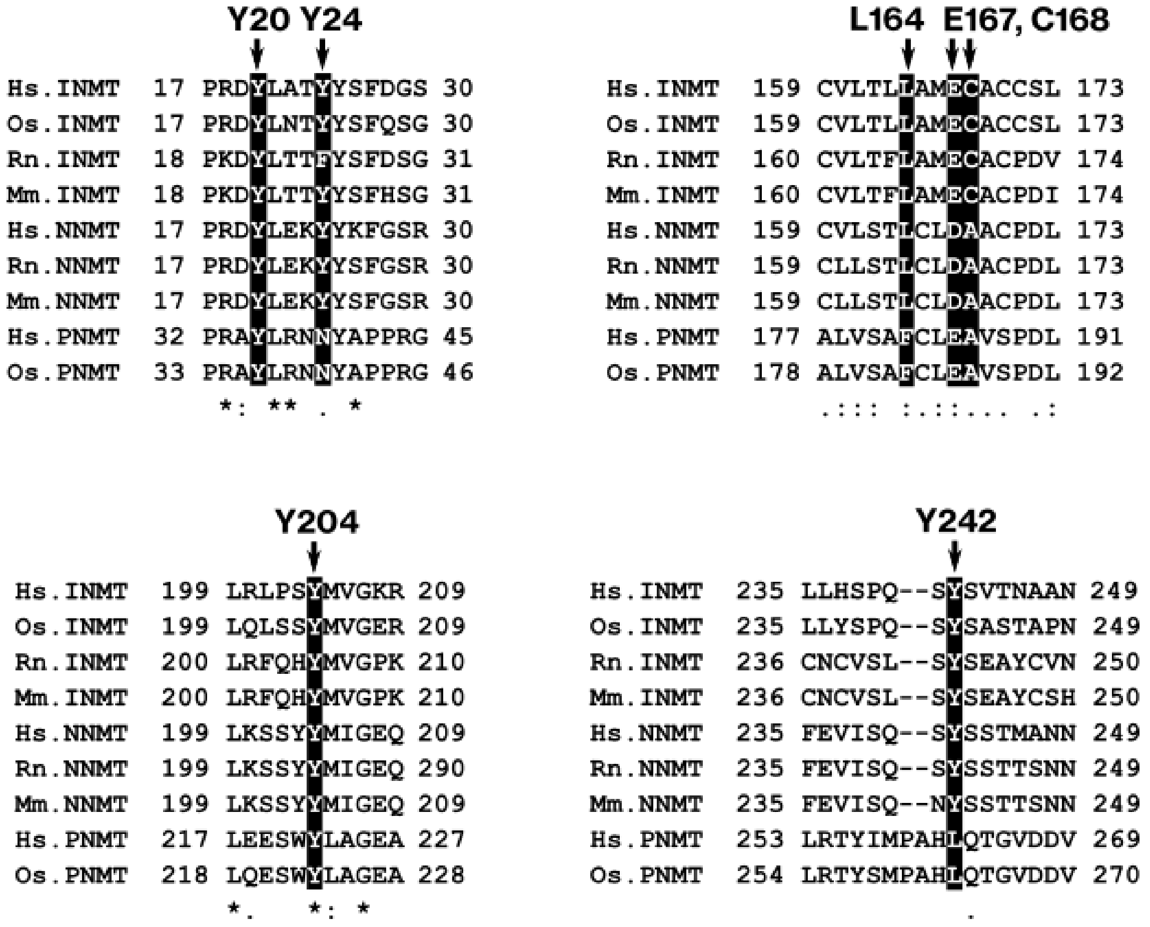

Figure S2. Multiple alignments of INMT, NNMT, and PNMT.

Amino acid sequences of INMT, NNMT, and PNMT were aligned using Clustal Omega. Amino acid residues that are predicted to interact with DMSe in the active center of INMT are highlighted in white letters on a black background. Specifically, human INMT residues Y20, Y24, L164, Y204, and Y242 are conserved in NNMT. Residues E167 and C168 in INMT are also indicated by arrows. Hs, human. Os, rabbit. Rn, rat. Mm, mouse.

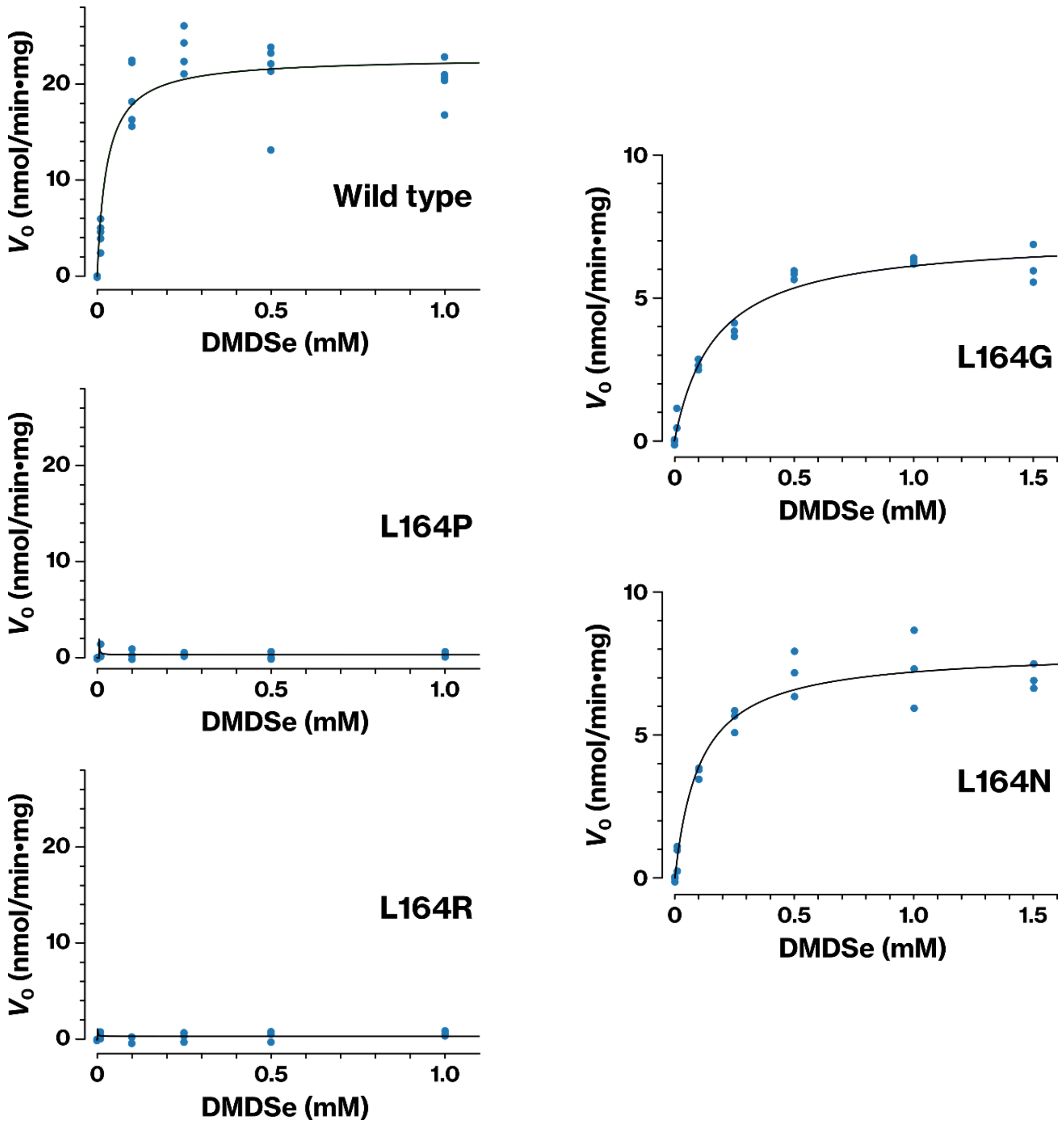

Figure S3. Quantitative analysis of *Se*-methyltransferase activity of INMT.

The methylation reaction of DMDSe by INMT was analyzed quantitatively. DMDSe was added to the methylation reaction at various concentrations, and the progress of the reaction was analyzed using the MTase-Glo™ methyltransferase assay kit. The Michaelis–Menten equation was fitted to the observed data using non-linear least square analysis. The plots represent results from more than three independent experiments. The corresponding V_{max} and K_M values are shown in Table 2.
